# A lightweight DeepME model based on improved YOLOv11 architecture for macular edema detection and treatment monitoring

**DOI:** 10.3389/fendo.2026.1864912

**Published:** 2026-07-03

**Authors:** Xue Bai, Ming Yi, Tianye Chen, Naijing Feng, Quan Shi, Tingxue Li, Rui Hua

**Affiliations:** 1 Department of Ophthalmology, The First Hospital of China Medical University, Shenyang, Liaoning, China; 2 The Research Center for Medical Genomics, Key Laboratory of Medical Cell Biology, Ministry of Education, School of Life Sciences, China Medical University, Shenyang, Liaoning, China; 3 Cardiopulmonary Rehabilitation Center, the Fourth People’s Hospital of Shenyang, China Medical University, Shenyang, Liaoning, China; 4 Suzhou Institute of Biomedical Engineering and Technology, Chinese Academy of Science, Suzhou, Jiangsu, China; 5 School of Automation and Electrical Engineering, Shenyang Ligong University, Shenyang, Liaoning, China

**Keywords:** anti-vegf, central foveal thickness, deep learning, diabetic macular edema, diabetic retinopathy, optical coherence tomography, swept-source OCT angiography, YOLOv11

## Abstract

**Objective:**

We developed and validated an improved YOLOv11-based deep learning algorithm for accurate macular edema detection in optical coherence tomography (OCT) images, and built DeepME—a lightweight system for diagnosis and treatment recommendations.

**Methods:**

We compiled a comprehensive dataset combining hospital clinical data and public OCT resources, covering macular edema and other retinal diseases. External validation used an anti-vascular endothelial growth factor (anti-VEGF) cohort of 336 eyes from 300 patients with diabetic retinopathy or retinal vein occlusion. The improved YOLOv11n integrated the Convolutional Block Attention Module (CBAM) to enhance feature extraction. DeepME combined this detector with an optimized DeepSeek model, current clinical guidelines, and expert knowledge.

**Results:**

DeepME achieved better performance over standard YOLOv11: accuracy 0.980, specificity 0.990, sensitivity 0.970, precision 0.990, F1-score 0.980, and AUC 0.9993. Grad-CAM visualizations confirmed precise localization of cystoid macular edema within anatomically correct retinal layers. In the anti-VEGF cohort, central foveal thickness decreased significantly at one-month follow-up (p < 0.001), and DeepME showed substantial agreement with manual grading (p < 0.001), enabling rapid, accurate diagnosis and treatment guidance.

**Conclusion:**

This study introduces DeepME, a novel clinical decision support system that integrates an improved YOLOv11 detection architecture for comprehensive macular edema management. DeepME delivers high accuracy in evaluating anti-VEGF treatment response and shows strong potential for real-world clinical decision support.

## 1 Introduction

Macular edema (ME) frequently occurs as a complication of diabetic retinopathy (DR) and retinal vein occlusion (RVO). Diabetic macular edema (DME), which arises from microvascular leakage in DR, is the leading cause of vision impairment in diabetic patients (1, 2). Globally, approximately 21 million individuals are affected by DME, with a prevalence rate ranging from 5.5% to 7.4% (3). Studies have demonstrated that among 22,896 diabetic patients, 10.2% exhibited DR, and 6.81% had DME. RVO ranks as the second most prevalent retinal vascular disease after DR, and RVO-ME serves as a primary target for RVO treatment (4, 5).

Intravitreal anti-vascular endothelial growth factor (anti-VEGF) injections currently represent the first-line therapy for DME and RVO-ME, effectively reducing macular edema and improving vision (6, 7). However, more than 30% of patients still exhibit persistent DME despite receiving regular intravitreal injections at least four times within 24 weeks (8). Additionally, some RVO-ME patients demonstrate insufficient responses to anti-VEGF drugs (5). These findings highlight the challenges associated with frequent follow-ups and repeated intravitreal injections in anti-VEGF treatment. Therefore, better tools with objective and stable full-course management are needed to optimize the diagnosis and treatment of macular edema.

Optical coherence tomography (OCT) is a non-invasive, high-resolution ophthalmic imaging technique that has become the gold standard for diagnosing and managing macular edema and other retinal diseases (9, 10). Swept-source OCT angiography (SS-OCTA) builds upon OCT by leveraging changes in light signals caused by blood flow to visualize vascular distribution and morphological alterations in tissues (11, 12). OCT and OCTA imaging play critical roles throughout the diagnostic and therapeutic processes of retinal-related diseases, serving not only as essential evidence for macular edema diagnosis but also as key tools for preoperative evaluations of anti-VEGF drug injections. However, the widespread application of OCT technology has generated a large volume of complex image data, imposing a significant burden on clinicians during image analysis. To address this challenge, several studies have integrated artificial intelligence (AI) with machine learning, deep learning, and other advanced methodologies, enable precise characterization of biomarker changes during disease progression and promote quantitative assessments. Based on ophthalmic imaging, AI plays a pivotal role in early detection, diagnostic grading, treatment evaluation, therapeutic recommendations, and prognostic prediction for common ophthalmic conditions. Toto et al. collected OCT images from DME patients and developed a YOLOv7 (You Only Look Once v7)-based model for detecting hard exudates, but it had low accuracy for small targets like microaneurysms (13). Miguel et al. proposed a hybrid CNN-RNN model using 22 pre-trained CNNs and bidirectional RNN layers, though it increased overfitting risks and required extensive data and resources (14). Jeribi et al. introduced a deep learning model based on CornerNet and DenseNet-100 for DR and DME detection, but it excluded OCT images and relied only on fundus images, potentially missing non-visible lesions (15). Consequently, developing an accurate and lightweight model for macular edema diagnosis remains our research objective. As the latest evolution in the YOLO series of object detection algorithms, YOLOv11 exhibits superior performance in detecting tiny lesions. Its lightweight architecture and robust anti-interference capability further solidify its potential for advancing imaging-related models.

Therefore, in this study, we aimed to develop and validate a novel deep learning algorithm based on an improved YOLOv11 architecture to enhance the detection accuracy of macular edema using OCT imaging. Furthermore, we designed a lightweight diagnostic and recommendation system, termed DeepME, to support the clinical management of patients with macular edema. This study has been reported in line with the TITAN Guidelines 2025 (16).

## 2 Materials and methods

The development of the algorithm section of our DeepME model.

### 2.1 Data sources and preprocessing

Our study constructed a dataset by integrating hospital clinical data (Department of Ophthalmology, The author’s Hospital: macular edema: 2410 OCT images; non-macular edema: 6010 OCT images) with online data from Kaggle (macular edema: 10,000 OCT images; non-macular edema: 6,000 OCT images), available at: https://www.kaggle.com/datasets/paultimothymooney/kermany2018. Hospital clinical data provided authentic and reliable cases, ensuring the dataset’s realism and clinical relevance. Online data enhanced sample diversity, addressing the limitations of relying solely on hospital data. There were no duplicate or overlapping cases between the two datasets. By integrating these two data sources, this study seeks to enhance the model’s diagnostic accuracy and adaptability, thereby facilitating early detection and precise treatment of ophthalmic diseases.

All patient DICOM data were rigorously anonymized prior to analysis to ensure compliance with GDPR and HIPAA regulations. The de-identification process consisted of the following steps: (1) removal of all protected health information (PHI), including patient names, identifiers, birth dates, and examination dates, performed using automated tools such as the DICOM Anonymizer Toolkit or validated in-house scripts; (2) all images were anonymized prior to analysis. Pixel-level anonymization was conducted using validated, in-house preprocessing scripts. For images containing patient-identifiable information embedded within anatomical regions, masking-based removal and region-specific filtering were applied to excise non-diagnostic textual or metadata elements—while rigorously preserving retinal anatomical structures and lesion-associated features; and (3) acquisition of institutional review board (IRB) approval for the use of retrospective data, with the informed consent requirement waived due to the fully anonymized nature of the dataset. This study adhered to the Declaration of Helsinki guidelines and was approved by the Ethics Committee of the author’s Hospital.

The dataset covers DME (6410 OCT images), and RVO-ME (6000 OCT images), and other eye diseases, including age-related macular degeneration (3010 OCT images), idiopathic choroidal neovascularization (3000 OCT images), epimacular membrane (3600 OCT images), and retinal detachment (2400 OCT images). This data fusion enhances the model’s generalization ability and diagnostic accuracy for various eye diseases. The complete dataset, comprising both the public Kaggle data and the internal hospital clinical data, was uniformly partitioned into training, validation, and test sets using a strict 8:1:1 ratio at the image level across all pathological classes, thereby ensuring a balanced proportional representation in the test set. Moreover, because fully anonymized public datasets lack patient identifiers, patient-level overlap within this subset cannot be definitively ruled out.

**Figure 1:**
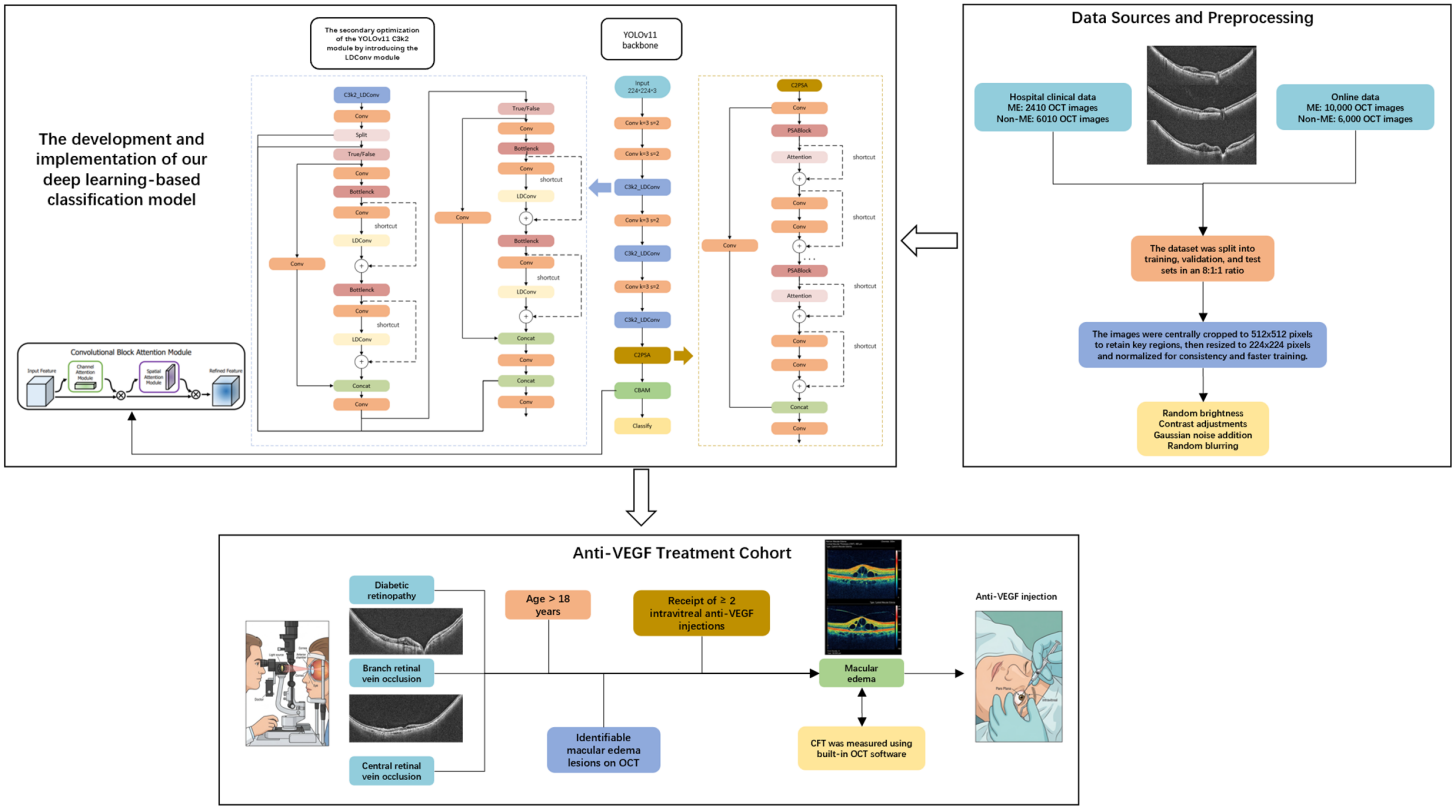
Flow chart of our study design.

For image preprocessing, hospital-acquired DICOM images and publicly available JPEG/PNG images exhibited substantial heterogeneity in native spatial resolution; therefore, the images were centrally cropped to 512×512 pixels to retain key regions, then resized to 224×224 pixels and normalized for consistency and faster training. Data augmentation techniques—including rotation, isotropic scaling, and horizontal flipping—were applied during training. Horizontal flipping was further leveraged to model right-eye and left-eye variations, as retinal OCT volumes from contralateral eyes typically exhibit approximate mirror symmetry in both anatomical architecture and lesion distribution. This strategy enhanced laterality diversity and mitigated ocular laterality bias within the dataset (**Figure 1**).

### 2.2 Macular edema diagnostic algorithm inspired by the architecture of YOLOv11n

Our diagnostic algorithm, based on the improved YOLOv11n, uses an efficient architecture to process large image datasets quickly and deliver real-time responses. It is ideal for applications requiring instant feedback, such as automated image classification, and maintains high speed and frame rates even on limited hardware. The algorithm integrates the Linear Deformable Conv (LDConv) module (17), updates the C3k2 module, and incorporates the Convolutional Block Attention Module (CBAM). We replaced the convolutional layers in the C3K2 module of the original YOLO11 with LDConv, and added the CBAM module after Block 7 and Block 9. The hyperparameters are batch=128, epochs=100, learning rate lr=0.01, and the optimizer is SGD. The parameter numbers of the original YOLOv11n and the modified model were recorded from the model summary for comparison. These enhancements improve accuracy and robustness in image classification while optimizing resource use. Multi-level feature extraction and enhancement modules further increase accuracy, and the lightweight design enables fast deployment and real-time performance. The algorithm handles complex conditions like occlusion and varying lighting, ensuring stable and reliable results across various hardware environments, especially on embedded systems and mobile devices (**Figure 1**).

### 2.3 Refinement of the C3k2 module

This study introduces a key innovation by applying the LDConv module to optimize the YOLOv11 C3k2 module. This enhances feature extraction flexibility and efficiency, improving adaptation to diverse inputs and hardware environments. The LDConv module (17) adaptively adjusts convolution kernel shape and size, increasing accuracy and robustness. It improves the model’s ability to capture complex and multi-scale features while optimizing resource use for stable performance across platforms. The modified C3k2-LDConv structure is illustrated in **Figure 1**.

### 2.4 Integration of convolutional block attention module attention mechanism

Our algorithm integrates the CBAM attention mechanism, which enhances model performance. CBAM includes two sub-modules: the Channel Attention Module and the Spatial Attention Module (18). This lightweight design reduces parameters, improves efficiency, and strengthens feature extraction from channels and spatial positions. The Channel Attention Module generates a 1D map to highlight important channels, while the Spatial Attention Module creates a 2D map to emphasize key spatial regions. Together, they help the network focus on critical information, improving accuracy and robustness in tasks like object detection and image recognition. **Figure 1** shows CBAM’s workflow.

### 2.5 Metrics for classification performance

To evaluate the classification method’s performance, we used accuracy, specificity, sensitivity, precision, and F1 score. The confusion matrix includes true positive (TP), true negative (TN), false positive (FP), and false negative (FN) as key indicators. $\text{Accuracy}=\frac{\text{TP}+\text{TN}}{\text{TP}+\text{FP}+\text{FN}+\text{TN}}$; $\text{Specificity}=\frac{\text{TN}}{\text{TN}+\text{FP}}$; $\text{Sensitivity}=\frac{\text{TP}}{\text{TP}+\text{FN}}$; $\text{Precision}=\frac{\text{TP}}{\text{TP}+\text{FP}}$; $\text{F}1\_\text{score}=\frac{2\text{TP}}{2\text{TP}+\text{FP}+\text{FN}}$

### 2.6 Confidence interval estimation

To quantify the uncertainty of point estimates, 95% confidence intervals (CIs) were calculated. For accuracy, specificity, sensitivity, and precision—metrics that represent binomial proportions—the Wilson score interval method was employed. This approach is preferred over the standard normal approximation for proportions, particularly when values are close to 0 or 1, as it yields more accurate coverage probabilities. For the F1-score, a harmonic mean of precision and recall, the confidence interval was approximated using the delta method (based on a first-order Taylor expansion), incorporating the covariance between these two correlated metrics to improve estimation accuracy.

### 2.7 Cross-entropy loss

Softmax Cross-Entropy Loss is usually used to optimize the probability distributions for each category.


$$L_{cls}=-\frac{1}{N}\sum_{i=1}^{N}\sum_{c=1}^{C}y_{i,c}log({\hat{y}}_{i,c})$$

*N* denotes the sample size.

*C* represents the number of categories.

*y_i,c_* is the true label (1 or 0, indicating whether a sample belongs to category [c]).

${\hat{y}}_{i,c}$ is the probability predicted by the model for category.

### 2.8 Software design

Building upon this foundation, we developed DeepME, which integrated an optimized DeepSeek language model (19) (DeepSeek-Medical-LLM-7B, v1.1), the latest Chinese clinical guidelines and consensus statements (20, 21), and expert clinical experience. Diagnostic reports are generated through a multi-module workflow. The YOLOv11-based DeepME (19) detection model is primarily responsible for lesion detection, localization, and classification from OCT/OCTA images. The detected lesion regions (“lesion images”) refer to the regions of interest automatically localized by the YOLO-based model from OCT or OCTA scans. PaddleOCR is used to extract structured textual or numerical information from image-associated examination data when available. VanillaNet serves as an auxiliary lightweight classification module for feature verification and rapid screening support.

The DeepSeek-Medical-LLM-7B v1.1 model does not directly analyze raw OCT images; instead, it receives structured outputs generated by the image-analysis modules, including lesion category, confidence score, retinal thickness-related measurements, and OCR-extracted information. The DeepSeek-Medical-LLM-7B v1.1 recommendation module was implemented using a hybrid prompt-guided and rule-constrained framework. Current Chinese clinical guidelines for DR and RVO, together with consensus recommendations for anti-VEGF treatment, were manually curated and converted into structured knowledge templates. These guideline-derived rules and expert-defined decision pathways were embedded into the system through predefined prompts and a rule-based knowledge module rather than through model fine-tuning.

For each case, the large language model (LLM) received structured outputs generated by the image-analysis modules, including lesion type, confidence score, retinal thickness-related measurements, and OCR-extracted examination information. These structured inputs were combined with guideline-based prompts that explicitly constrained the generation process to evidence-supported diagnostic interpretations and treatment recommendations. The model therefore functioned as a clinical decision-support assistant that generated recommendations based on predefined clinical knowledge and structured imaging findings, rather than as an autonomous diagnostic system.

**Figure 2:**
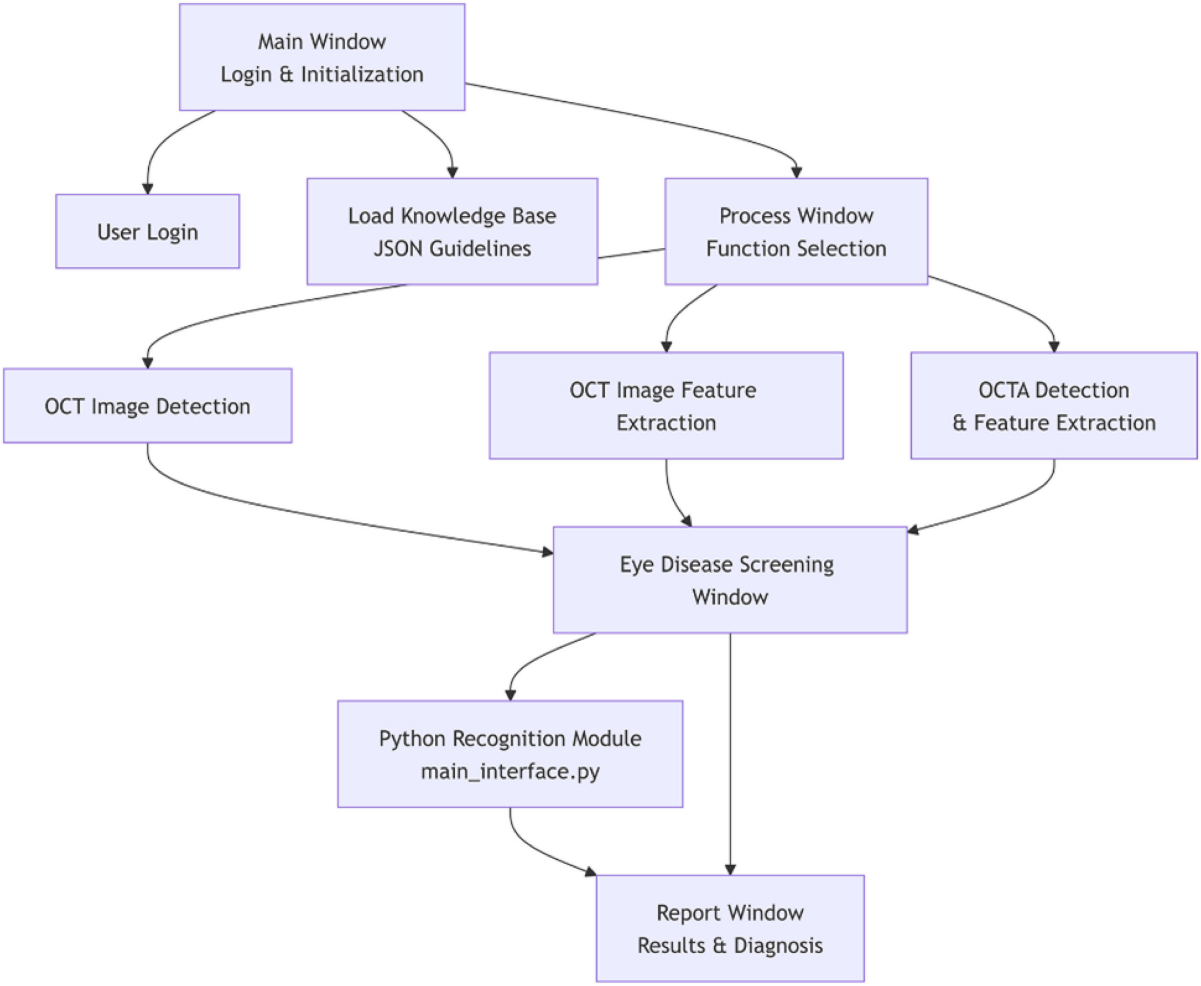
Software design flowchart.

**Figure 3:**
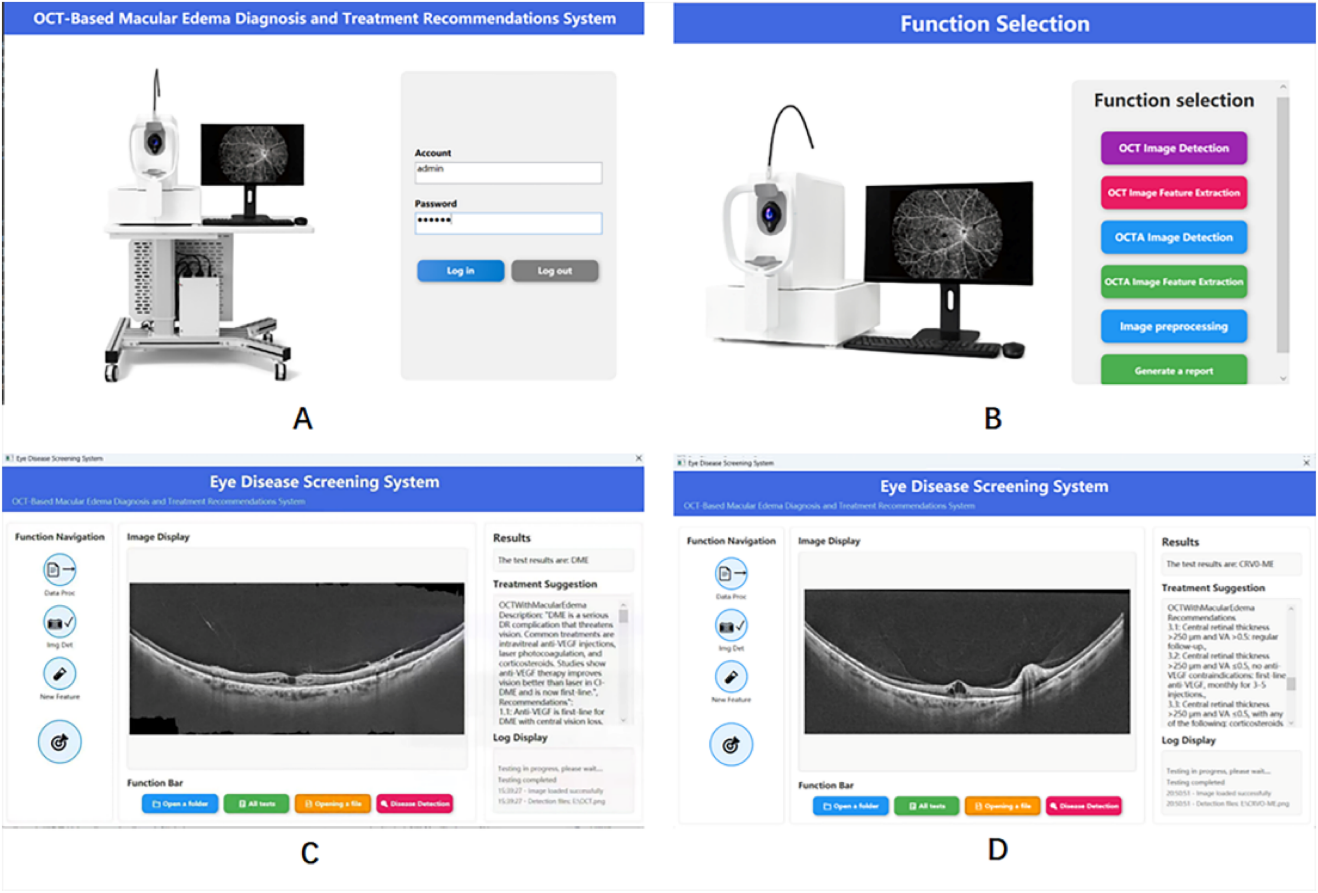
Implementation of the DeepME system. **(A)** Login Interface Design. **(B)** Function Selection Interface Design. **(C)** Disease Screening and Report Output for DME Case. **(D)** Disease Screening and Report Output for CRVO-ME Case.

The software platform includes three major functional pathways after function selection: (1) OCT Image Detection, (2) OCT Image Feature Extraction, and (3) OCTA Detection and Feature Extraction, which are consistent with the workflow illustrated in **Figures 2**, **3**. The final diagnostic recommendation is generated through the integration of multimodal imaging features and structured clinical information under physician supervision.

### 2.9 Anti-VEGF treatment cohort as an external validation

Patients diagnosed with macular edema due to DR, branch RVO, or central RVO between June 2019 and January 2025, were enrolled consecutively without selection bias to ensure representativeness. Eligibility criteria included: age > 18 years; visible macular edema on Spectral domain (SD)-OCT (SPECTRALIS OCT2, Heidelberg Engineering, Germany) or Swept source (SS)-OCT (VG200D, Intalight, China); and ≥ 2 intravitreal anti-VEGF injections, including aflibercept (2.0 mg/0.05 mL, Bayer), conbercept (0.5 mg/0.05 mL, Kanghong), or ranibizumab (0.5 mg/0.05 mL, Novartis) (22). Patients were excluded if macular edema resulted from other causes such as age-related macular degeneration, uveitis, or Irvine-Gass syndrome, or if images were unreadable due to severe vitreous or retinal hemorrhage. This cohort’s data was acquired in a non-overlapping temporal window relative to the training set, thereby satisfying the requirement for temporal external validation.

Baseline data, including sex, age, eye type, and drug used, were collected. Central foveal thickness (CFT) was measured using OCT software. Anti-VEGF drugs (ranibizumab, aflibercept, or conbercept) were administered under a 3+ PRN regimen (**Figure 1**). All injections were performed by the same ophthalmologist (X.B.), with drug selection based on clinical need and patient preference. Macular edema on OCT B-scan images was assessed manually by two ophthalmologists (TY.C. and X.B.) and compared with results from our DeepME model.

To ensure an objective comparison and minimize assessment bias, a blinded evaluation protocol was implemented. Two ophthalmologists (TY.C. and X.B.) independently reviewed the OCT B-scan images without access to the DeepME model’s predictions. Their assessments were based exclusively on visual inspection and clinical judgment. When disagreement occurred, Dr. Rui Hua conducted a blinded re-evaluation using standardized criteria and key OCT features: cystoid spaces and retinal thickening. If discordance remained, a three-member expert panel reviewed the case—incorporating clinical history and supplementary tests—to reach a final diagnosis. All steps were documented; the two original ophthalmologists jointly confirmed the diagnosis, and the attending clinician retained ultimate responsibility. The evaluations were then compared with the automated outputs of the DeepME model, which were generated without human intervention during the prediction phase. This blinded design effectively minimized the risk of AI output influencing the manual grading process.

### 2.10 Statistical analysis

Data were analyzed using SPSS (v22.0, IBM Corp., USA). Normally distributed variables were reported as mean ± standard deviation. Paired t-tests compared CFT before and after anti-VEGF treatment. The agreement between the DeepME and clinical assessments of macular edema was evaluated using the kappa coefficient, along with its 95% CIs. Cohen’s kappa values were interpreted as follows: ≤ 0.00, no agreement; 0.01–0.20, minimal to slight; 0.21–0.40, fair; 0.41–0.60, moderate; 0.61–0.80, substantial; and 0.81–1.00, nearly perfect agreement (23). A p-value < 0.05 was considered statistically significant. The methods for calculating 95% CIs for classification performance metrics are detailed in the ‘Confidence Interval Estimation’ subsection above.

## 3 Results

### 3.1 Diagnostic results of our DeepME model

**Figure 4:**
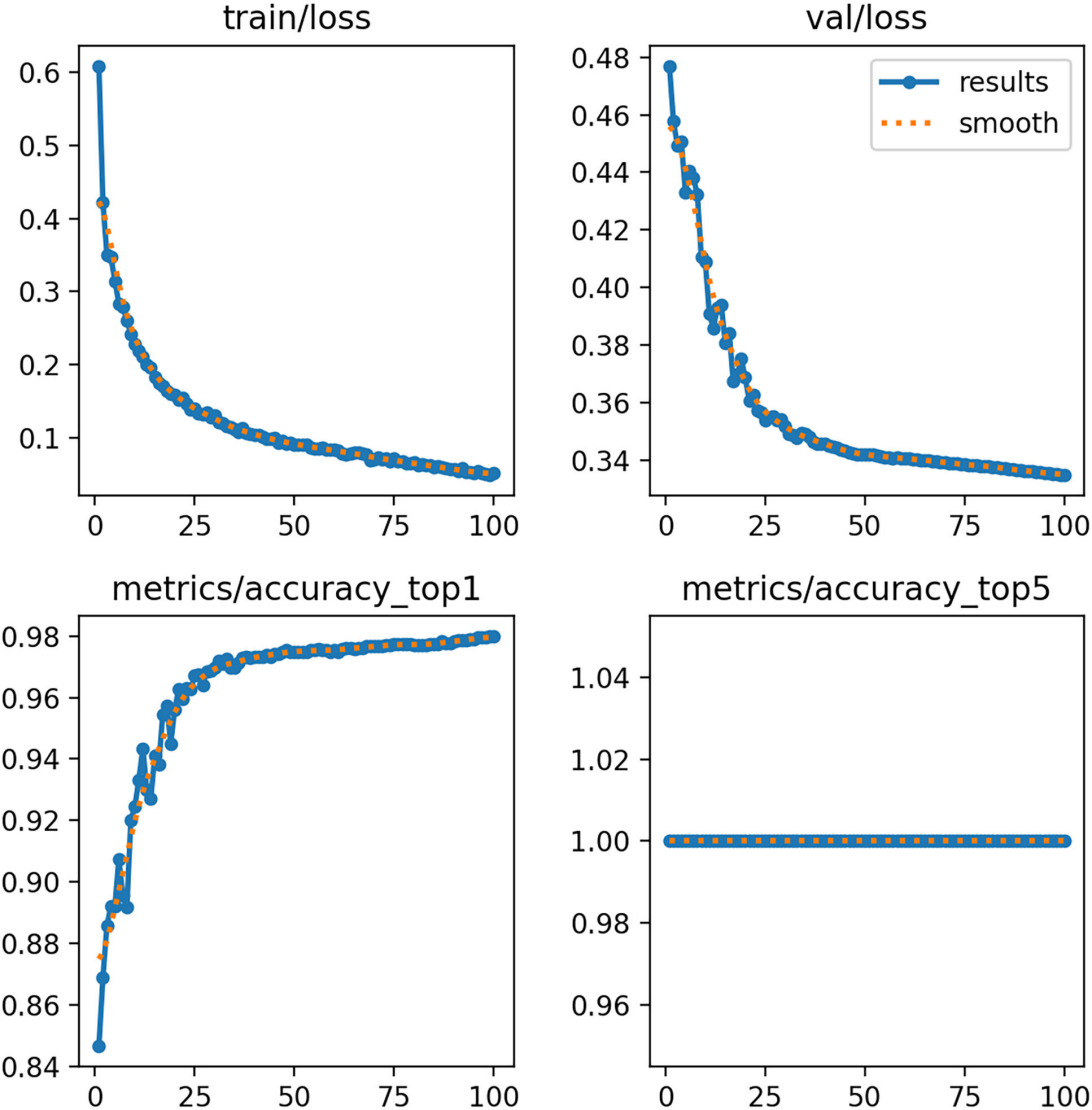
Training performance of the DeepME model over 100 epochs. The upper-left and upper-right subplots display the loss curves for the training and validation sets, respectively. As the number of training epochs increases, the loss values steadily decrease and eventually converge. The lower-left subplot shows the model’s Top-1 accuracy, which gradually improves and stabilizes at approximately 0.980 during training. The lower-right subplot presents the Top-5 accuracy, consistently maintained at 1.0 throughout the training process, demonstrating the model’s high recognition accuracy.

**Figure 5:**
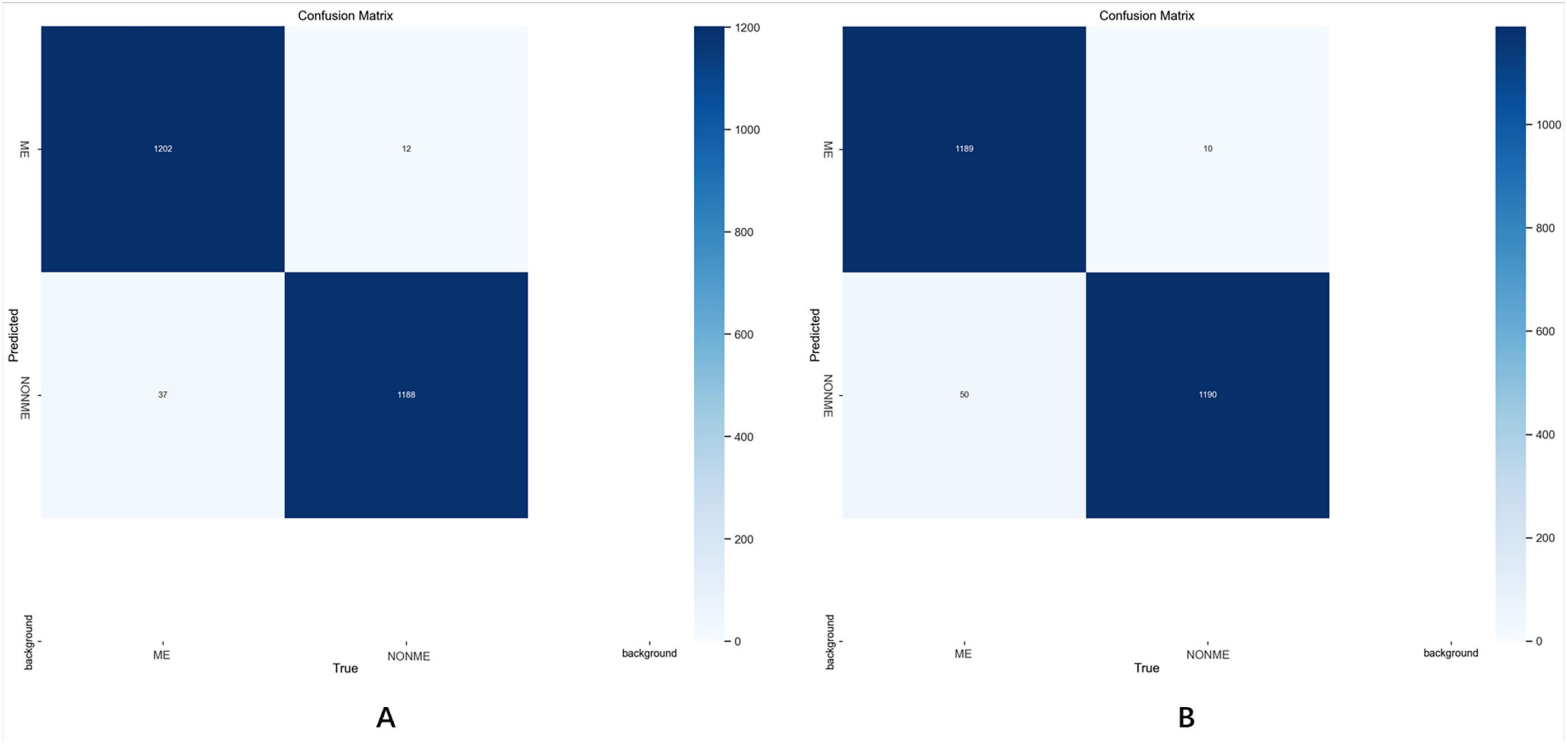
The confusion matrix of the classification results. **(A)** DeepME model; **(B)** YOLOv11. The TP count in the upper left corner, indicates the number of samples correctly identified as macular edema. The FN count in the lower left corner, represents samples that are truly macular edema but were incorrectly classified as non-macular edema. The FP count in the upper right corner, denotes samples that are actually non-macular edema but were misclassified as macular edema. Finally, the TN count in the lower right corner, indicates the number of samples correctly identified as non-macular edema. Agreement with manual judgment was high for both DeepME (κ=0.960, 95% CI 0.949–0.971) and YOLOv11 (κ=0.951, 95% CI 0.939–0.963; both *p* < 0.001).

The experiments were conducted on a system equipped with an NVIDIA GeForce RTX 5090 GPU, an Intel Core Ultra 9 285K processor, and 128 GB of RAM. The learning rate was set to 0.01, with stochastic gradient descent (SGD) as the optimizer, a batch size of 128, and a training schedule of 100 epochs. The training results are presented in **Figure 4**. The resulting confusion matrix is presented in **Figure 5**, providing a detailed breakdown of the model’s classification performance. Based on the confusion matrix (true positives = 1202, false negatives = 37, false positives = 12, true negatives = 1188; total N = 2439), the model demonstrated high performance, with accuracy = 0.980 (95% CI: 0.974–0.985), sensitivity (recall) = 0.970 (95% CI: 0.961–0.980), specificity = 0.990 (95% CI: 0.985–0.995), precision = 0.990 (95% CI: 0.985–0.995), and F1-score = 0.980 (95% CI: 0.973–0.986). The narrow confidence intervals reflect precise and stable estimates of the model’s diagnostic capability.

### 3.2 Performance comparison

**Figure 6:**
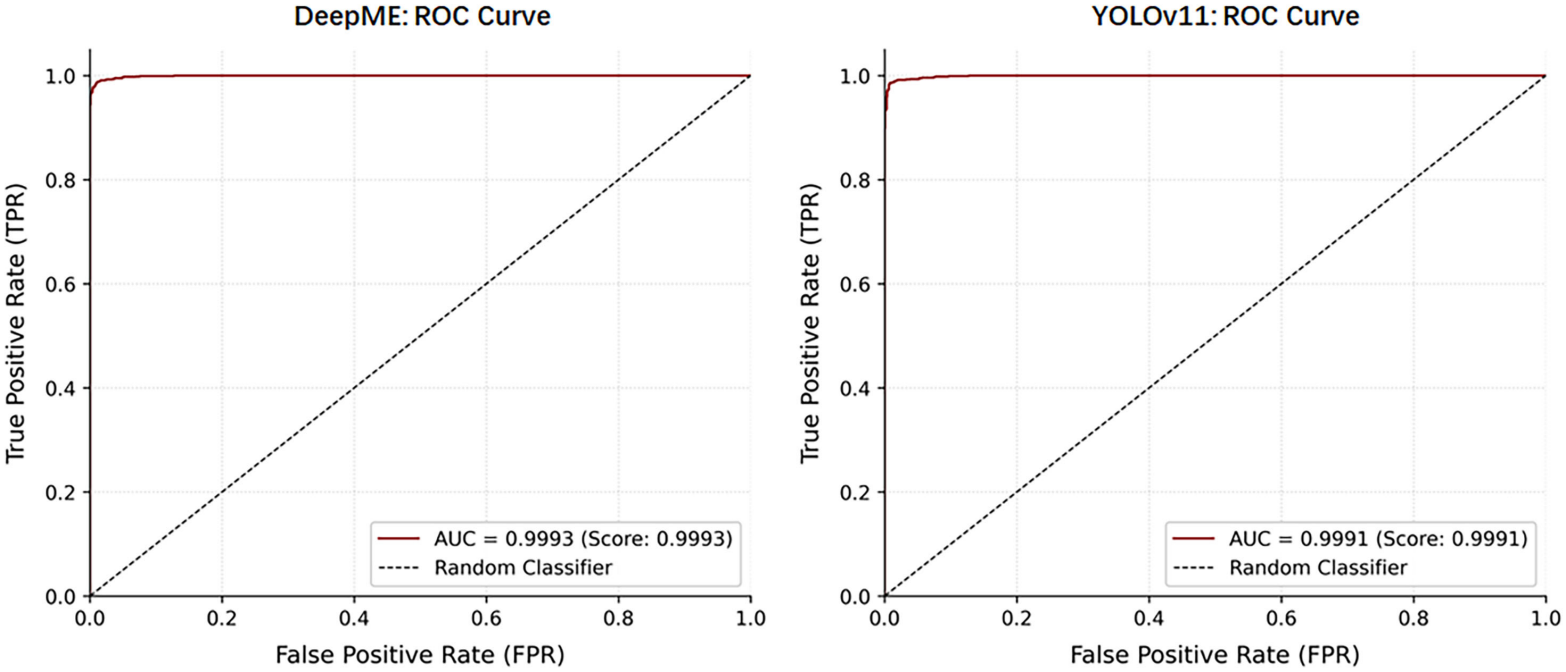
Presents the ROC curves and corresponding AUC values of DeepME and YOLOv11. The solid red line represents the ROC curve of the proposed classification model, reflecting its classification performance, while the dashed line indicates the performance of a random classifier serving as the baseline. On the left is the ROC curve of DeepME, with an area under the curve (AUC) of 0.9993; on the right is that of the standard YOLO model, with an AUC of 0.9991. Both curves are closely positioned to the upper-left corner of the plot, indicating strong lesion classification capability in both models. The improved DeepME model showed a marginally higher AUC than the standard YOLO model (0.9993 vs. 0.9991); this difference should be interpreted cautiously because statistical comparison of AUCs was not performed.

**Figure 7:**
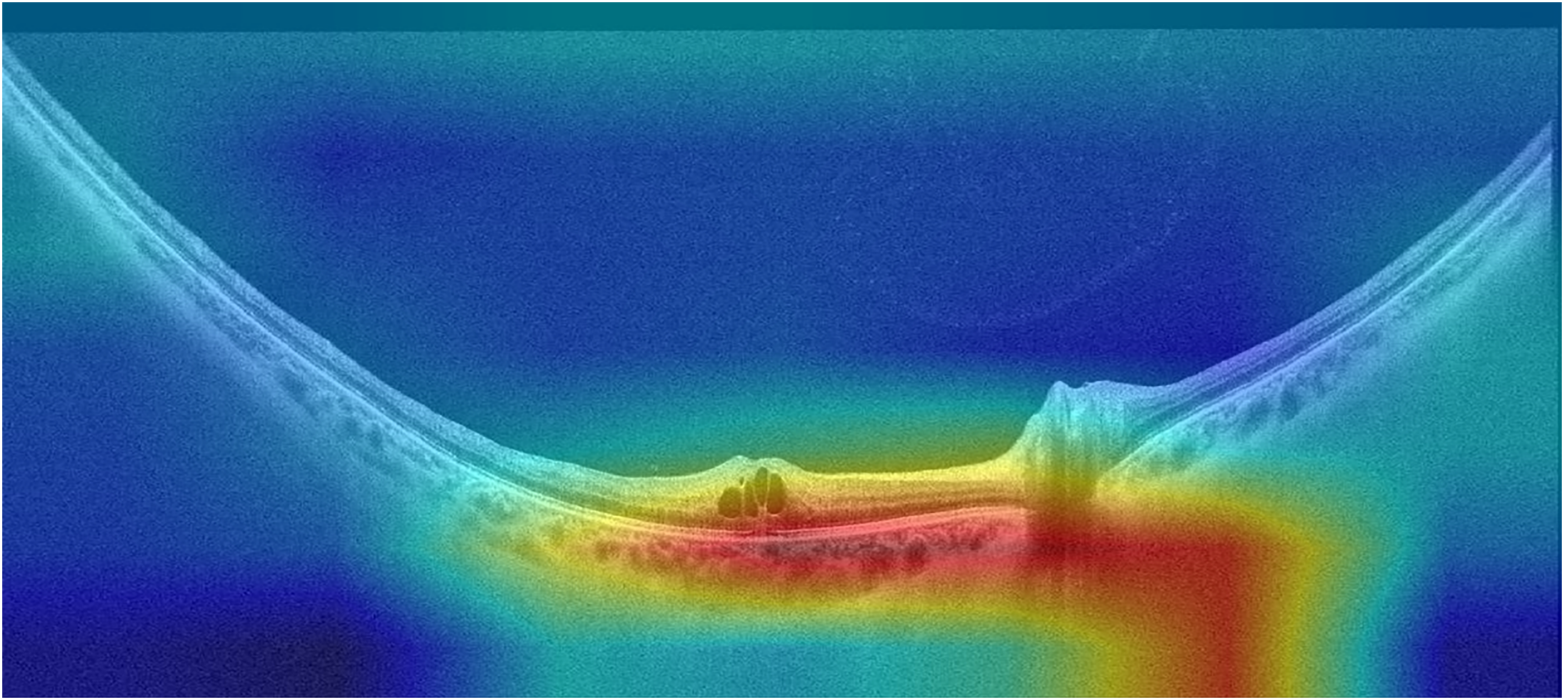
The class activation mapping (CAM) heat map generated by the DeepME deep learning model. The color gradient ranges from blue to red, representing increasing levels of model attention across different regions. The red and yellow highlighted areas are concentrated in the typical lesion sites of macular edema within the OCT image, visually illustrating the key visual features that the model utilizes for lesion identification. This not only confirms the medical rationality of the model’s decision-making process but also assists clinicians in rapidly localizing suspicious lesions and integrating AI-generated outputs to support more accurate diagnostic assessments.

This study systematically compared the performance of two structurally distinct deep learning models—DeepME and YOLO11—in the task of classifying ME versus non-ME cases (**Table 1**). All models were trained using identical data preprocessing and training protocols, ensuring a fair and consistent comparison. After 100 training epochs, both models achieved remarkably high classification accuracy and robustness. Notably, DeepME outperformed YOLO11 in discriminative ability, achieving an area under the ROC curve (AUC) of 0.9993 (**Figure 6**). Grad-CAM-based heatmap visualization further revealed that DeepME consistently localized cystoid edema lesions within retinal layers on OCT images with high spatial precision (**Figure 7**). This finding not only substantiates the effectiveness of the CBAM attention mechanism in prioritizing critical pathological regions during feature extraction but also highlights the model’s strong interpretability. As a result, DeepME offers a reliable and transparent decision-support tool for clinical diagnosis, potentially enhancing the accuracy and confidence of ophthalmic assessments.

**Table 1:** Performance metrics.

Model | performance metrics	Accuracy	Sensitivity	Specificity	Precision	F1 score
YOLOv11	0.975 (0.970,0.982)	0.960 (0.950,0.972)	0.992 (0.988,0.998)	0.992 (0.988,0.998)	0.975 (0.969,0.981)
DeepME model	0.980 (0.974,0.985)	0.970 (0.961,0.980)	0.990 (0.985,0.995)	0.990 (0.985,0.995)	0.980 (0.973,0.986)

95% confidence intervals (in parentheses) were calculated using the Wilson score interval method for accuracy, sensitivity, specificity, and precision, and the delta method for the F1-score for each model.

### 3.3 DeepME’s performance in evaluating macular edema following anti-VEGF therapy

This study included 336 eyes from 300 patients with macular edema. The mean patient age was 56.8 ± 11.31 years, comprising 152 males and 148 females. Among the eyes, 104 had branch RVO, 104 had central RVO, and 128 had DME. Treatments consisted of aflibercept (64 eyes), conbercept (52 eyes), and ranibizumab (220 eyes). Baseline mean CFT was 489.8 ± 219.18 µm. All CFT measurements were normally distributed according to the Shapiro-Wilk test (p = 0.12). All patients completed one-month follow-up after initial anti-VEGF treatment. Mean CFT significantly decreased from 489.8 ± 219.18 µm at baseline to 304.4 ± 143.24 µm at follow-up (t = 15.240, p < 0.001). DeepME showed excellent agreement with manual assessment for detecting macular edema, with a kappa value of 0.735 (95% CI: 0.668–0.802; p < 0.001).

### 3.4 Development and implementation of our DeepME system

The system workflow includes three stages (**Figure 3**): 1. Login and Initialization (Main Window): User authentication and knowledge base loading. 2. Function Selection (Process Window): Centralized access to OCT detection, feature extraction, and preprocessing tools. 3. Disease Screening and Report Output (Eye Disease Screening Window): Image recognition, lesion classification, and structured diagnostic report generation. This workflow ensures seamless interaction between the WPF-based user interface and Python-based recognition modules (3.8.18), offering clinicians an efficient, accurate, and scalable diagnostic platform.

## 4 Discussion

This study developed a novel DeepME system, integrated with a deep learning algorithm based on the improved YOLOv11, an optimized DeepSeek language model, the latest clinical guidelines and consensus statements, and expert clinical experience. While the application of attention augmented object detection models is established in medical imaging, the primary innovation of our work lies in constructing a closed-loop clinical decision support system. DeepME bridges the gap between high precision anatomical lesion detection via OCT and automated, guideline based clinical reasoning to create the paradigm from images to treatment. To enhance model generalization and reduce bias from single-center data, this study built a comprehensive dataset combining clinical data from the Ophthalmology Department of the author’s Hospital and Retinal OCT Images from Kaggle (https://www.kaggle.com/). These data were used to train and validate the OCT-based detection of macular edema. We propose Window Normalization (WIN), which uses local window statistics to adaptively normalize features, and combine it with WIN-WIN self-distillation to significantly improve out-of-distribution generalization on heterogeneous medical imaging data (24). Since dataset quality directly affects model performance, this study ensured clinical relevance and diversity by combining real-world hospital data with online resources. Additionally, an anti-VEGF treatment cohort was used to test the model. Patients followed a 3+PRN injection protocol, and their OCT scans were evaluated by ophthalmologists and compared with results from the new algorithm. The results showed substantial agreement between DeepME and manual assessments, demonstrating significant improvements in both diagnostic accuracy and generalization ability of the proposed DeepME model.

Our DeepME innovatively applied the YOLOv11n to the diagnosis of macular edema for the first time. Compared with standard YOLOv11, the improved YOLOv11 algorithm exhibited better performance in detecting OCT images of macular edema, significantly enhancing sensitivity to minor lesions and resolving false positive issues caused by noise or low contrast in OCT images. Traditional OCT image analysis primarily relies on classic ResNet and standard YOLOv11. ResNet optimizes deep network training through residual connections and is mainly used for grading diabetic retinopathy and macular edema (25, 26). However, the global average pooling layer of ResNet compresses spatial features, easily misclassifying minor lesions and surrounding tissues as noise (27). Moreover, ResNet requires additional introduction of 3D convolution or LSTM for 3D dynamic detection, increasing computational complexity (28). YOLO reconstructs target detection as a regression problem, predicting bounding box coordinates and class probabilities directly from the complete image through a single neural network (29). YOLOv11 is one of the latest evolved versions of the YOLO series of target detection models, capable of real-time target detection and suitable for dynamic monitoring, optimizing computational efficiency (30). However, standard YOLOv11 struggles to precisely identify lesion areas due to mismatched default anchor box sizes in medical images. To address the drawbacks of traditional algorithms, we enhanced the YOLOv11 deep learning framework by integrating the CBAM attention mechanism. CBAM improves the network’s focus on critical information through two complementary components: the channel attention module and the spatial attention module. As a lightweight and versatile module, CBAM can be seamlessly embedded into any CNN architecture, effectively highlighting target regions and enhancing classification and detection accuracy across models (17, 18). Its integration systematically boosted overall model performance, particularly for complex image features, improving computational efficiency and enabling deployment on resource-constrained platforms without sacrificing real-time performance. We further optimized the C3k2 module by incorporating the LDConv module, which adaptively adjusts convolution kernel shape and size to enhance feature extraction accuracy and robustness (17). To improve model robustness, data augmentation techniques such as brightness and contrast adjustment, Gaussian noise addition, and random blurring were applied during training. Through analysis and comparison of OCT images of macular edema using the same dataset, the results confirmed that our improved DeepME model outperformed YOLOv11 in comprehensive scores.

Both DeepME and the baseline YOLOv11 achieved exceptional, near-ceiling performance on this classification task (AUC = 0.9993 vs. 0.9991). At this performance tier, the absolute difference of 0.0002 is statistically indistinguishable and clinically non-actionable—reflecting metric saturation rather than model superiority. Consequently, the principal clinical value of DeepME resides not in marginal discriminative gains, but in two functionally integrated advances: (i) enhanced interpretability via CBAM-augmented Grad-CAM visualizations that demonstrate precise anatomical alignment with pathological lesions (**Figure 7**), thereby supporting clinician verification and trust; and (ii) its operational design as an end-to-end clinical decision-support system that unifies lesion detection with LLM-generated, guideline-concordant treatment recommendations. The difference between DeepME and the baseline YOLOv11 should be interpreted as a descriptive improvement rather than definitive statistical superiority. In population-scale screening programs, even marginal enhancements in sensitivity can substantially reduce false-negative rates, thereby improving diagnostic reliability and aligning with the stringent performance requirements of primary healthcare settings (31). Furthermore, Grad-CAM–based heatmap visualization confirmed that DeepME consistently localized cystoid macular edema lesions within anatomically defined retinal layers on OCT images, with high spatial fidelity. DeepME achieves highly consistent lesion localization with expert clinicians on OCT images, accurately delineating the anatomical boundaries of macular edema lesions across diverse cases. This robust spatial fidelity enables rapid, reliable differentiation of macular edema from phenotypically similar macular pathologies—especially for less-experienced primary care clinicians—improving diagnostic accuracy and differential diagnosis efficiency. Grad-CAM–enhanced models boost lesion recognition with anatomy-aligned visual explanations, reducing missed and false-positive diagnoses in resource-limited or high-throughput settings (32). CAM heatmaps support rapid lesion localization for large-scale DME screening (33). Grad-CAM validates predictions by precisely highlighting DME-affected retinal regions—confirming pathophysiological plausibility and minimizing reliance on expert annotation (34). The model rapidly processes OCT images significantly accelerating the diagnostic workflow compared to traditional manual slice by slice review. When deployed in primary healthcare settings, it can enhance macular edema screening efficiency and reduce the burden on tertiary hospitals. The algorithm maintained stable performance under occlusion and varying lighting conditions. Even on low-resource hardware, it achieved high inference speed and frame rate, improving classification reliability.

Furthermore, the DeepME model was designed not only to enhance OCT image analysis accuracy but also to integrate multiple complementary components into a comprehensive clinical decision support system for macular edema management. The system incorporated an optimized version of the DeepSeek language model, enabling precise interpretation of medical literature, patient records, and clinical notes. This allowed the system to analyze complex clinical cases and deliver context-aware recommendations. The latest Chinese clinical guidelines and consensus statements on macular edema were also embedded into the system’s knowledge base, ensuring all diagnostic and treatment suggestions aligned with current standards of care. By combining these evidence-based guidelines with expert clinical experience, DeepME could simulate high-level clinical reasoning. The platform features a lightweight architecture, making it suitable for deployment in diverse clinical settings, including primary care centers with limited computing resources. This diagnostic and recommendation tool supports OCT-based macular edema detection and generates personalized follow-up and treatment plans, improving patient management efficiency and healthcare outcomes. It is essential to emphasize that the proposed AI system is designed to assist, rather than replace, clinical decision-making. Model outputs should be interpreted as advisory inputs for qualified physicians, who retain full responsibility for diagnostic and therapeutic decisions. Clinicians retain final authority over all clinical decisions and full accountability for patient outcomes. AI outputs—such as diagnostic suggestions, treatment options, and risk assessments—must be reviewed, validated, and integrated with clinical expertise before use in patient care (35).

The DeepME recommendation module delivers guideline-concordant decision support—not independent clinical judgment. It embeds current clinical guidelines and expert pathways via structured prompts and rule-based templates, ensuring recommendations align with evidence. However, the module was not validated against expert treatment plans. Its outputs are therefore clinician-facing aids—not validated autonomous recommendations. Future work will assess safety, reliability, and clinical utility through expert-panel review and prospective real-world evaluation. To mitigate potential medical liability risks, we recommend integrating a mandatory physician review into clinical workflows. Furthermore, future implementations should incorporate explainability features—such as saliency maps or uncertainty quantification—to enhance transparency, facilitate clinical interpretation, and build trust in AI-assisted systems.

Our study used three anti-VEGF drugs—Aflibercept, Conbercept, and Ranibizumab—all of which inhibit pathological vascular changes driven by VEGF. However, this therapy carries risks such as over-treatment and difficulties in long-term monitoring. The DeepME model effectively addresses these challenges. The optimized YOLOv11 algorithm enables seamless integration of “Images–Algorithms–Treatment,” allowing dynamic monitoring of OCT changes before and after anti-VEGF injections. By tracking the entire treatment course, the model helps optimize injection frequency and reduce the number of required procedures. However, there was limited research on resistance to anti-VEGF treatment or disease recurrence. Extending follow-up time and collecting longer-term OCT data will help verify the robustness of DeepME in dynamic monitoring. Moreover, our external validation cohort was relatively small, a limitation that should be addressed in future research. For the development of the DeepME model, the dataset comprising 24,420 OCT images was partitioned into training, validation, and test sets according to an 8:1:1 ratio. The anti-VEGF treatment cohort was enrolled consecutively between June 2019 and January 2025 without selection bias to ensure representativeness for evaluating the clinical application value of the DeepME model. Despite its relatively small sample size, the cohort retains adequate representativeness and statistical power. While DeepME achieved agreement on the internal test set (kappa = 0.960), we observed a substantial drop in performance on the external validation cohort (kappa = 0.735). This was primarily attributable to the image-level (rather than patient-level) dataset split, which—particularly for the public dataset—precluded complete exclusion of patient-level overlap. Consequently, internal performance metrics approached ceiling levels, in contrast to those observed in the external validation cohort. Moreover, this discrepancy also highlights a generalization gap likely caused by domain shift. The external cohort included images from different hardware modalities. Specifically, both SD-OCT (Heidelberg Engineering) and SS-OCT (Intalight) which introduce variations in image contrast, resolution, and artifact profiles. Furthermore, the anatomical alterations following repeated anti-VEGF injections present unique morphological challenges compared to treatment-naïve lesions. Therefore, future prospective studies with diverse device representations are required to against hardware induced domain shifts. Owing to the strict irreversible anonymization of the retrospective hospital data and the inherent lack of patient metadata in the public online dataset, we were unable to assess potential demographic confounders (e.g., age, gender, diabetes status) across the training and testing splits. Future prospective studies should incorporate comprehensive clinical metadata to further validate the model’s robustness against demographic variations. Currently, this system is intended for research purposes only. Clinical deployment would require additional validation and the integration of a human-in-the-loop mechanism to ensure patient safety, regulatory compliance, and accountability.

## 5 Conclusion

In this study, a new AI model was developed using deep learning algorithms based on the advanced YOLOv11 architecture, improving the detection rate of macular edema in OCT images. The algorithm was further applied to monitor OCT-based prognosis following anti-VEGF treatment. Based on these capabilities, a novel clinical decision-support model named DeepME was developed. It enables dynamic OCT monitoring and supports personalized treatment planning, offering a scientifically grounded and efficient approach for managing ophthalmic diseases. Furthermore, it demonstrates strong potential to guide clinical decision-making in the management of macular edema.

## Data Availability

The original contributions presented in the study are included in the article/supplementary material. Further inquiries can be directed to the corresponding author.
